# Supplementary material for: CalCORVID: a dynamic RShiny dashboard approach to visualize spatiotemporal clusters for public health surveillance
Source: BMC Public Health. 2026 Jan 13;26:925. doi: 10.1186/s12889-026-26201-1 (PMC12997703; doi:10.1186/s12889-026-26201-1)
Supplement: Supplementary file 1 — Supplementary Material 1. [file 12889_2026_26201_MOESM1_ESM.docx]

## Supplementary Information


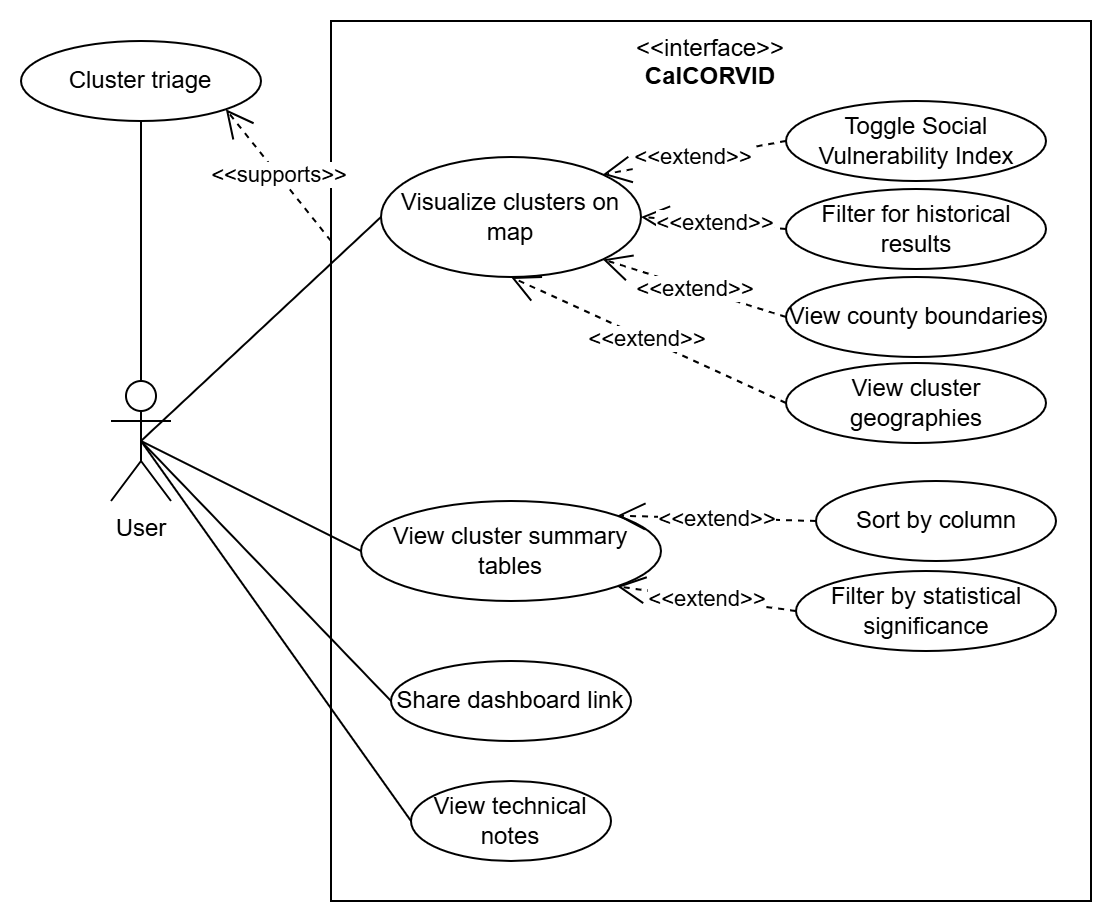


**Supplementary Figure 1.   Use case diagram for the CalCORVID dashboard showing primary user interactions in applied public health settings. Each oval represents a distinct use case, capturing a specific task or goal that users can perform through the dashboard interface. The diagram includes <<extend>> to indicate optional or conditional features and <<supports>> to show enabling processes that contribute to core functionality. The dashboard enables users to explore detected clusters, compare attributes, share a website link with relevant stakeholders, and view technical notes related to the model. These interactions help inform cluster triaging decisions.**


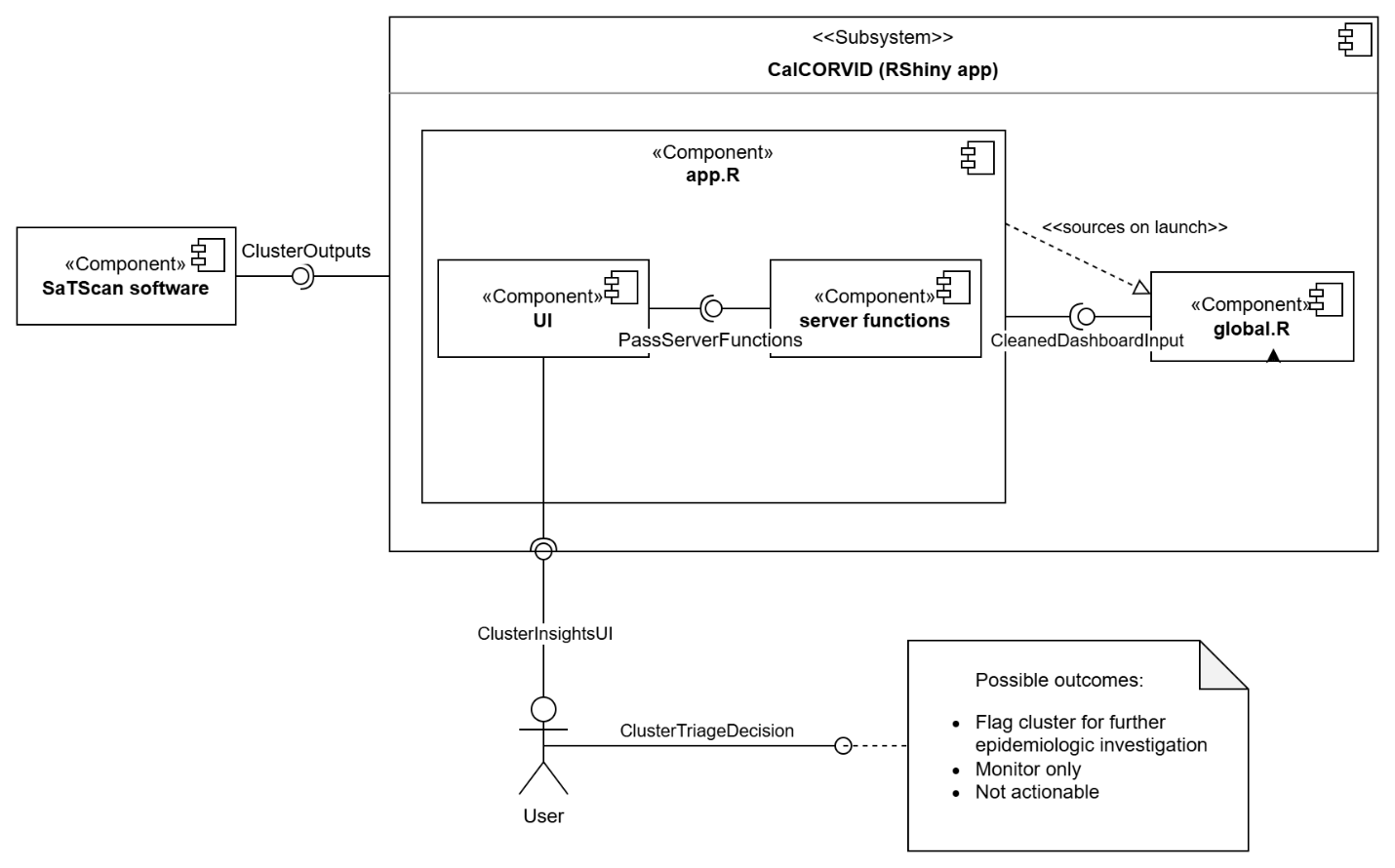


**Supplementary Figure 2.  Component diagram showing the key inputs, components, and outputs of the CalCORVID dashboard. The app.R component is the entry point of the application and automatically sources the global.R component at runtime. global.R cleans and formats the ClusterOutputs generated outside the dashboard, exposing a provided interface (ball symbol) that is required by app.R (hinge symbol). Once the cleaned dataset is returned, app.R uses its server logic (PassServerFunctions) and UI definitions to render the dashboard interface (ClusterInsightsUI). The user can then use the dashboard interface to triage cluster results outside the dashboard (ClusterTriageDecision).**

###### Supplementary Table 1. Software and R packages used to develop the CalCORVID dashboard.

| **Name and version** | **Type** | **Description** | **Purpose/Relevance** | **Depends and Imports (required R packages)** |
| --- | --- | --- | --- | --- |
| R (4.0.4) | Software | Language and environment for statistical computing and graphics (1) | Programming language the dashboard is based off of | — |
| SaTScan (10.1) | Software | Free software that analyzes spatial, temporal, and spatiotemporal data using the space-time scan statistic (2) | User must obtain results from SaTScan to adapt dashboard code | — |
| dplyr (1.1.3) | R package | A fast, consistent tool for working with data frame like objects, both in and out of memory (3) | Manipulate and clean data to display on the dashboard | cli, generics, glue, lifecycle, magrittr, methods, pillar, R6, rlang, tibble, tidyselect, utils, vctrs |
| DT (0.18) | R package | Data objects in R can be rendered as HTML tables using the JavaScript library 'DataTables' (typically via R Markdown or Shiny) (4) | Generate table to display cluster results on dashboard | htmltools, htmlwidgets, httpuv, jsonlite, magrittr, crosstalk, jquerylib, promises |
| findSVI (0.1.2) | R package | Provided with year(s), region(s) and a geographic level of interest, 'findSVI' retrieves required variables from US census data and calculates SVI for communities in the specified area based on Centers for Disease Control/Agency for Toxic Substances and Disease Registry (CDC/ATSDR) SVI documentation (5) | Obtain Social Vulnerability Index (SVI) metrics displayed in cluster tooltip | cli, dplyr, magrittr, purrr, stringr, tidycensus, tidyr, tidyselect, rlang, utils |
| leaflet (2.0.4.1) | R package | Create and customize interactive maps using the ‘leaflet’ Javascript library and ‘htmlwidgets’ package (6) | Render map for overlaying cluster results in dashboard | crosstalk, htmltools, htmlwidgets, jquerylib, leaflet.providers, magrittr, methods, png, raster, RColorBrewer, scales, sp, stats, viridisLite, xfun |
| lubridate (1.7.10) | R package | Functions to work with date-times and time-spans, including parsing date-time data and algebraic manipulation of date-time and time-span objects (7) | Obtain dates at the beginning of a time frame (e.g., week start date) | methods, generics, timechange |
| rsatscan (1.0.7) | R package | Functions to write R data frames into SaTScan-readable formats, set SaTScan parameters, and run SaTScan software using a wrapper (8) | Run to obtain sample data | utils, foreign |
| sf (0.9.8) | R package | Support for simple features, a standardized way to encode spatial vector data (9) | Read in the shapefile containing county boundaries to toggle as an optional layer; calculate centroids for map zoom levels | methods, classInt, DBI, graphics, grDevices, grid, magrittr, Rcpp, s2, stats, tools, units, utils |
| shiny (1.7.1) | R package | A web application framework to build interactive web applications with R (10) | Underlying framework to develop RShiny dashboard | methods, utils, grDevices, httpuv, mime, jsonlite, xtable, fontawesome, htmltools, R6, sourcetools, later, promises, tools, crayon, rlang, fastmap, withr, commonmark, glue, bslib, cachem, lifecycle |
| shinyjs (2.1.0) | R package | Perform common useful JavaScript operations in Shiny apps that will greatly improve your apps without having to know any JavaScript (11) | Enable clicking images on the home page to switch tabs | digest, jsonlite, shiny |
| shinythemes (1.2.0) | R package | Themes for use with Shiny (12) | Set dashboard theme | shiny |
| stringr (1.5.0) | R package | A consistent, simple and easy to use set of wrappers around the fantastic 'stringi' package (13) | Use for text string matching to identify CSV files | cli, glue, lifecycle, magrittr, rlang, stringi, vctrs |
| tigris | R package | Downloads TIGER/Line shapefiles from the United States Census Bureau and load into R as ‘sf’ objects (14) | Obtain county boundaries, state coordinates to orient leaflet map, and obtain polygons for clusters | stringr, magrittr, utils, rappdirs, httr, uuid, sf, dplyr, methods |

######

###### Supplementary Table 2. Model specification used in SaTScan software to generate the sample display data. Default values were used for any unspecified parameters.

| **[Setting] Parameter** | **Parameter Setting** |
| --- | --- |
| [Input] Study Period | September 21, 2021-February 8, 2022 |
| [Input] Coordinates | Lat/Long |
| [Analysis] Analysis type | Prospective space-time |
| [Analysis] Probability Model | Discrete Poisson |
| [Analysis] Scan For areas With: | Low rates |
| [Analysis] Time Aggregation | Units: Day, Length: 7 |
| [Analysis 🡪 Spatial Window Shape] Circular or Elliptic | Circular |
| [Analysis→Spatial Window] Maximum Spatial Cluster Size | 50 km |
| [Analysis→Temporal Window] Maximum Temporal Cluster Size | 40 days |
| [Analysis→Temporal Window] Minimum Temporal Cluster Size | 1 day |
| [Analysis→Cluster Restrictions] Restrict low rate clusters to observed/expected less than or equal to: | 0.10 |

**Supplementary Table 3. A summary table of the differences between CalCORVID and other existing SaTScan-related RShiny dashboards. CalCORVID differs from SpatialEpiApp and EpiExploreR in that it provides a flexible dashboard framework focused on visualizing and disseminating SaTScan results. In contrast, SpatialEpiApp and EpiExploreR are designed to simplify the execution of SaTScan models. Additionally, CalCORVID is open source, which allows the dashboard code to evolve and be adapted over time. SpatialEpiApp and EpiExploreR do not provide open-source code and are therefore more static in their design and functionality.**

|  | **CalCORVID** | **SpatialEpiApp** | **EpiExploreR** |
| --- | --- | --- | --- |
| **Purpose** | Flexible RShiny dashboard for displaying and sharing SaTScan results, with added interactivity and support for external data like the Social Vulnerability Index (SVI) | Combines two common approaches in health surveillance, disease mapping and cluster detection, into a singular interface that does not require programming (15) | Combines multiple spatiotemporal analytic packages into a singular interactive interface, developed specifically for rapidly analyzing and visualizing data on animal diseases in Italy (16) |
| **Supported statistical approaches** | SaTScan | SaTScan, R-INLA | SaTScan, EpiCurve, EpiVelocity, tpaths |
| **Contains a SaTScan software wrapper** | No; requires user to run SaTScan outside of the dashboard to obtain outputs | Yes; required to use built-in wrapper to display results but provides a limited subset of parameter options | Yes; required to use built-in wrapper to display results but provides a limited subset of parameter options |
| **Supported SaTScan models** | All prospective space-time probability models, but specifically designed for the discrete Poisson and space-time permutation models | Only provides “spatial” and “spatiotemporal” options; no option to specify probability model so is unclear | Space-time permutation |
| **Required input/s** | A comma separated value (CSV) file containing SaTScan model results with columns described in Table 1 | Shapefile of study area, CSV file of cases containing area ID, date, population, and cases | Unclear; there are built-in datasets and there appears to be an option to upload your own dataset |
| **Availability** | Open source code repository available on Github at github.com/cdphmodeling/CalCORVID | Public app is available at paulamoraga.shinyapps.io/spatialepiapp | Public app link no longer works, ongoing and future support unclear |

## Supplementary References

1. R: The R Project for Statistical Computing [Internet]. [cited 2024 Jan 22]. Available from: https://www.r-project.org/

2. SaTScan - Software for the spatial, temporal, and space-time scan statistics [Internet]. [cited 2024 Jan 22]. Available from: https://www.satscan.org/

3. Wickham H, François R, Henry L, Müller K, Vaughan D, Software P, et al. dplyr: A Grammar of Data Manipulation [Internet]. 2023 [cited 2024 Jan 22]. Available from: https://cran.r-project.org/web/packages/dplyr/index.html

4. Xie Y, Cheng J, Tan X, Allaire JJ, Girlich M, Ellis GF, et al. DT: A Wrapper of the JavaScript Library “DataTables” [Internet]. 2023 [cited 2024 Jan 22]. Available from: https://cran.r-project.org/web/packages/DT/index.html

5. Xu [aut H, cre, cph. findSVI: Calculate Social Vulnerability Index for Communities [Internet]. 2023 [cited 2024 Jan 30]. Available from: https://cran.r-project.org/web/packages/findSVI/index.html

6. Cheng J, Schloerke B, Karambelkar B, Xie Y, Wickham H, Russell K, et al. leaflet: Create Interactive Web Maps with the JavaScript “Leaflet” Library [Internet]. 2023 [cited 2024 Jan 22]. Available from: https://cran.r-project.org/web/packages/leaflet/index.html

7. Spinu V, Grolemund G, Wickham H, Vaughan D, Lyttle I, Costigan I, et al. lubridate: Make Dealing with Dates a Little Easier [Internet]. 2023 [cited 2024 Jan 22]. Available from: https://cran.r-project.org/web/packages/lubridate/index.html

8. Kleinman K, Hostovich S, Moosa A. rsatscan: Tools, Classes, and Methods for Interfacing with “SaTScan” Stand-Alone Software [Internet]. 2023 [cited 2024 Feb 13]. Available from: https://cran.r-project.org/web/packages/rsatscan/index.html

9. Pebesma E, Bivand R, Racine E, Sumner M, Cook I, Keitt T, et al. sf: Simple Features for R [Internet]. 2023 [cited 2024 Jan 22]. Available from: https://cran.r-project.org/web/packages/sf/index.html

10. Chang W, Cheng J, Allaire JJ, Sievert C, Schloerke B, Xie Y, et al. shiny: Web Application Framework for R [Internet]. 2023 [cited 2024 Jan 22]. Available from: https://cran.r-project.org/web/packages/shiny/index.html

11. Attali [aut D, cre. shinyjs: Easily Improve the User Experience of Your Shiny Apps in Seconds [Internet]. 2021 [cited 2024 Jan 30]. Available from: https://cran.r-project.org/web/packages/shinyjs/index.html

12. Chang W, RStudio, themes) TP (Bootswatch, font) LD (Lato, font) NW (News C, fonts) GC (Open S and R, et al. shinythemes: Themes for Shiny [Internet]. 2021 [cited 2024 Feb 6]. Available from: https://cran.r-project.org/web/packages/shinythemes/index.html

13. Wickham H, Software P, PBC. stringr: Simple, Consistent Wrappers for Common String Operations [Internet]. 2023 [cited 2024 Jan 22]. Available from: https://cran.r-project.org/web/packages/stringr/index.html

14. Walker K, Rudis B. tigris: Load Census TIGER/Line Shapefiles [Internet]. 2024 [cited 2024 Jul 8]. Available from: https://cran.r-project.org/web/packages/tigris/index.html

15. Moraga P. SpatialEpiApp : A Shiny web application for the analysis of spatial and spatio-temporal disease data. Spat Spatio-Temporal Epidemiol. 2017 Nov;23:47–57.

16. Savini L, Candeloro L, Perticara S, Conte A. EpiExploreR: A Shiny Web Application for the Analysis of Animal Disease Data. Microorganisms. 2019 Dec;7(12):680.
